# Preparation and Optimization of Bovine Serum Albumin Nanoparticles as a Promising Gelling System for Enhanced Nasal Drug Administration

**DOI:** 10.3390/gels9110896

**Published:** 2023-11-13

**Authors:** Sandra Aulia Mardikasari, Gábor Katona, Bence Sipos, Rita Ambrus, Ildikó Csóka

**Affiliations:** 1 Institute of Pharmaceutical Technology and Regulatory Affairs, Faculty of Pharmacy, University of Szeged, H-6720 Szeged, Hungary; sandraaulia@unhas.ac.id (S.A.M.); sipos.bence@szte.hu (B.S.); ambrus.rita@szte.hu (R.A.); csoka.ildiko@szte.hu (I.C.); 2 Faculty of Pharmacy, Hasanuddin University, Makassar 90245, Indonesia

**Keywords:** bovine serum albumin (BSA), desolvation method, ethanol, gelation, nasal drug delivery

## Abstract

Bovine serum albumin (BSA) has been used extensively as a suitable carrier system for alternative drug delivery routes, such as nasal administration. However, the optimization of BSA nanoparticles with respect to their nasal applicability has not been widely studied. The present study focuses on the characterization of BSA nanoparticles prepared using the desolvation method, followed by a gelation process to facilitate intranasal drug delivery. The results demonstrated that the ratio of BSA and the desolvating agent, ethanol, played a critical role in the nanoparticle characteristics of the BSA nanogel matrices (BSA-NGs). Based on the gelling properties, the formulations of BSA-NG 2, BSA-NG 4, and BSA-NG 6 were selected for further investigation. The Raman spectra confirmed that there were no specific changes to the secondary structures of the BSA. The mucoadhesion studies revealed moderately high mucoadhesive properties, with a mucin binding efficiency (MBE) value of around 67%, allowing the dose to avoid elimination due to rapid mucociliary clearance of the nasal passage. Via studying the nexus of the carrier system, BSA-NGs loaded with dexamethasone as a model drug were prepared and evaluated by differential scanning calorimetry (DSC) and thermal gravimetry (TG), ascertaining that no ethanol remained in the samples after the freeze-drying process. Furthermore, the viscosity measurements exhibited moderate viscosity, which is suitable for nasal liquid preparations. The in vitro release studies performed with a simulated nasal electrolyte solution (SNES) medium showed 88.15–95.47% drug release within 4 h. In conclusion, BSA nanoparticle gelling matrices can offer potential, value-added drug delivery carriers for improved nasal drug administration.

## 1. Introduction

In the last few decades, nanomedicine has gained focus regarding pharmacological research and development in order to improve the effectiveness of drug therapy. Various nanocarriers (polymeric, lipid-, and protein-based) are tested nowadays that offer numerous attractive features, including the possibility of enhancing bioavailability and utilizing them for alternative drug delivery routes. Among the various protein-based carrier systems, the albumin-based systems show remarkable features. Albumin has garnered considerable interest due to its beneficial properties, including biocompatibility, biodegradability, non-immunogenicity, ease of availability, and lack of toxicity [1]. Furthermore, albumin also possesses various physicochemical properties that are beneficial in supporting drug delivery formulations, such as its ability to enhance water solubility, stability, and permeability. Via proper formulation control, various drug release profiles and, most importantly, controlled release can be achieved [2,3,4].

The application of albumin in drug delivery has been widely investigated, including for nasal delivery purposes, and appears a promising method to successfully deliver drugs through the nasal cavity with the desired mucoadhesive properties and permeability at the site of action [5,6,7]. Albumin nanoparticles can be prepared using several methods, such as desolvation or coacervation, emulsification, thermal gelation, self-assembly, nab^®^ technology, and some other techniques [8,9]. The desolvation method is known to be the most frequently used method and offers a simple way of fabricating nanoparticles by employing a desolvating agent (i.e., ethanol) to perform protein denaturation; subsequently, the gelation process yields albumin nanoparticles [10]. However, the proper ratio of albumin and ethanol to produce the appropriate gel matrices with the relevant characteristics for nasal route application has not been widely explored.

Furthermore, bovine serum albumin (BSA) has emerged as one of the most frequently investigated serum albumins for drug delivery [11]. BSA originates from bovine serum and has almost the same properties as human serum albumin (~80% amino acid sequence homology) [4], but it also has a relatively low cost and is easy to purify, which makes it more widely used in research [12]. BSA is a highly water-soluble protein with a molecular weight of approximately 69 kDa and comprises around 585 amino acids [4,11]. BSA shows stability at a pH range of 4–9, corresponding to the pH value of alternative drug delivery routes and their dosage form requirements, and it can be heated for ten hours at 60 °C without denaturation [13].

The present study aimed to prepare and optimize the fabrication of BSA nanogel matrices (BSA-NGs) through ethanol-induced BSA hydrogel formation or via desolvation methods and then investigate the obtained BSA-NG properties corresponding to the nasal conditions. At first, the optimization of BSA-NGs was conducted by preparing several formulations with various ratios of BSA, ethanol, and purified water. Afterward, characterization of the prepared BSA-NGs was carried out for the average hydrodynamic diameter (Z-average), polydispersity index (PdI), and zeta potential (ZP), as well as the viscosity, pH, and mucoadhesive properties in accordance with nasal applicability. After that, the folding behavior of BSA-NGs was investigated by measuring the change in the secondary structure of BSA due to ethanol addition via Raman spectroscopy. Finally, the drug release profiles were compared with the rheological properties of the BSA-NG formulations and then studied using dexamethasone (DXM) as a model drug, which is used as a supplementary steroidal anti-inflammatory agent for the local treatment of upper respiratory tract infections such as acute bacterial sinusitis.

## 2. Results and Discussion

### 2.1. Optimization of BSA Nanoparticle Gel Matrices

#### 2.1.1. Results of the Factorial Design

The optimization of the BSA-NG formulation through the ethanol-induced gelation process was investigated using a three-factor, three-level factorial design with nine standard runs. The ratio of all variables and the obtained responses of the Z-average, PdI, and ZP values are shown in Table 1. Using the TIBCO Statistica 13.4 software, the polynomial equations were created to describe the individual main effects, as well as the interaction effects between the independent variables on each dependent variable. Moreover, the Z-average and PdI are highly influenced by experimental conditions. Therefore, the responses of these two parameters were employed for this optimization. Successful nanogel formulation requires the preparation of homogenous (polydispersity index PDI < 0.7) nanocarriers with a Z-average of 50–200 nm [14]. The surface plots are shown in Figure S1 in the Supplementary Materials.

According to the multiple regression analysis of the experimental data, the relationship of the independent variables on the Z-average (*Y*_1_) can be described using the following equation:

(1) $$Y_{1}={99.61+16.995x}_{1}+{14.985x}_{2}-{22.1x}_{3}+12.2325x_{2}^{2}-27.235x_{3}^{2}-13.89x_{1}x_{2}+16.11x_{1}x_{2}^{2}$$

The regression coefficient (*R*^2^) of the surface plot was 0.98086. Based on the polynomial equation, the positive sign before the variables indicates that the effects of those variables are directly proportional to the Z-average. The increase in the concentration of BSA and ethanol subsequently increases the value of the Z-average. The interaction between BSA and ethanol can encourage gelation to occur, thus generating the larger particle size of BSA-NG [10,15,16,17]. Meanwhile, the negative sign indicates that the effect of the variable is inversely proportional to the Z-average. The increase in purified water results in a reduced particle size of BSA-NG. The reduction of the Z-average value could be due to the ability of purified water to enhance the solubilization of BSA, leading to a lower concentration of BSA in the formulation for performing gelation while in contact with ethanol [18].

Furthermore, the effect of the independent variables on the PdI (*Y*_2_) can be described with the following equation:

(2) $$Y_{2}={0.475444+0.0275x}_{1}-{0.029x}_{3}-0.024583x_{1}^{2}+0.011417x_{2}^{2}+0.094583x_{3}^{2}-0.259833x_{1}x_{2}$$

The regression coefficient (*R*^2^) of the surface plot was 0.98228. It was found that the amount of water (*x*_3_) and the interaction coefficient of BSA and ethanol (*x*_1_*x*_2_) demonstrated a significant effect on the PdI. The coefficients with positive signs indicate the directly proportional relationship of the variable with the PdI, which means that a higher amount of the variable can lead to a higher PdI value in the resulting formulation. The higher PdI value can be explained as being due to the increased concentration of BSA affecting the tendency of the particles to form aggregates [19]. Moreover, the negative sign of the coefficients indicates that the effect of the variables is inversely proportional to the PdI, in which the addition of those variables might decrease the PdI value. The amount of purified water influences the concentration of BSA in the formulation, in terms of the ability to solubilize BSA, resulting in a reduced aggregation of the particle and generating a lower PdI value [19].

#### 2.1.2. pH Measurement

The pH of the BSA-NG preparations was investigated to confirm their applicability for use in accordance with nasal pH conditions. Importantly, pH is a parameter for nasal preparations that influences patient compliance when using the preparation, and the pH can significantly affect the nasal ciliary beat frequency [20]. Therefore, it is of great importance for the pH of the preparation to be within set parameters for nasal application.

The results of the pH measurement showed that the pH values for nine preparations of BSA-NG were in the range of 6.7 to 7.0, which indicates the non-irritating behavior of the BSA-NG and appears appropriate for nasal utilization. In addition, the pH of human nasal mucosa is typically around 5.5–6.5 [21], with a baseline of about ~6.3 [22]; significantly, the pH might appear to change with slight changes in the patient’s illness and condition, which is in the range of 5.3–7.6 [23].

Furthermore, considering the gelling appearances of all formulations after the gelation occurred, in which three different formations were observed (a bluish opalescent soft gel, liquid gel, and hard gel), and taking into account the variation of ethanol amounts in the formulations (0.6, 0.9, and 1.2 mL), the formulations of BSA-NG 2, BSA-NG4, and BSA-NG 6 were selected for further investigation.

### 2.2. Raman Spectroscopic Structural Investigation

**Figure 1:**
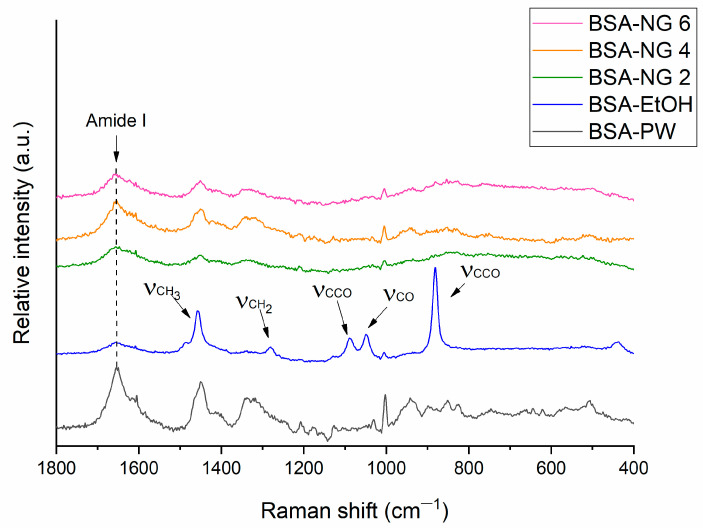
Raman spectrum of the BSA aqueous solution (BSA-PW), BSA nanoparticles after being desolvated with ethanol (BSA-EtOH), and BSA-NGs after the freeze-drying process (BSA-NG 2, BSA-NG 4, and BSA-NG 6).

The addition of ethanol to the BSA solution by up to 30% *v*/*v* can cause the unfolding of albumin, indicating a change in the α-helical structure. Therefore, Raman spectroscopic studies were performed (Figure 1) to investigate the refolding behavior of BSA-NGs after the removal of ethanol through the freeze-drying process.

The intensity changes of the peak at 1655 cm^−1^ (amide I region) in the Raman spectra of BSA, which can usually be attributed to the ordered *α*-helix (*ho*), indicate the unfolding and refolding of the protein. In the Raman spectrum of BSA-EtOH, the decreased intensity of the amide I peak suggests the unfolding of BSA [24], while the peaks appearing at 882 cm^−1^ (CCO skeleton symmetric stretching vibration), 1047 cm^−1^ (CO scaling), 1088 cm^−1^ (CCO skeleton stretching vibration), 1279 cm^−1^ (CH_2_ deformation), and 1456 cm^−1^ (CH_3_ antisymmetric deformation) are characteristic of ethanol [25]. These characteristic peaks cannot be identified in the spectrum of the BSA nanogel, indicating a successful freeze-drying process, while the increased intensity of the amide I peak in comparison to BSA-EtOH supports the possible partial refolding of BSA after the redispersion of the nanogel in purified water, due to hydrophobic interactions.

### 2.3. Mucoadhesion Studies

**Figure 2:**
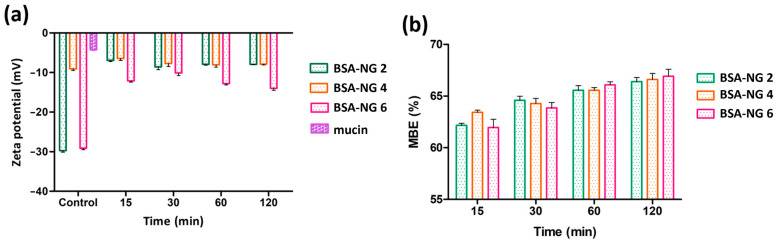
Mucoadhesive behavior of BSA NG preparations (BSA-NG 2, BSA-NG 4, and BSA-NG 6) with the zeta potential measurement method (**a**) and the turbidimetric method (**b**).

The evaluation of mucoadhesive properties of the selected BSA-NGs (BSA-NG 2, BSA-NG 4, and BSA-NG 6) was performed using two different methods: the ZP measurement method and the turbidimetric method. In the ZP method, the results showed that the BSA-NGs (BSA-NG 2, BSA-NG 4, and BSA-NG 6) demonstrated an observable alteration in their ZP values after incorporation with mucin solution, as depicted in Figure 2a. The changes in ZP values can be explained by the interaction of BSA and the mucin solution, leading to the formation of mucin-BSA complexes, a process that is primarily driven by electrostatic interactions, especially with a highly negatively charged molecule [26,27]. In addition, BSA is known to actively bind with mucin and the interactions are markedly higher at a neutral pH [26]. Accordingly, in this case, since all formulations of BSA-NGs were at a neutral pH, with a relatively high negative charge (BSA-NG 2 and BSA-NG 6), higher electrostatic interactions can substantially take place to promote the formation of mucin–BSA complexes, which can be observed through the alteration of ZP in both BSA-NG 2 and BSA-NG 6. Meanwhile, in the case of BSA-NG 6, the changes in ZP were observed with a slightly lower negative charge, possibly due to a higher ratio of purified water being used in the formulation. Consequently, it led to a reduction in the electrostatic forces between the molecules (BSA-NG 4 and mucin), enabling the formation of hydrogen bonds and van der Waals interactions [27].

Furthermore, the turbidimetric method, also known as the indirect method, highlighted an increased mucin binding efficiency (MBE) for all BSA-NG preparations (BSA-NG 2, BSA-NG 4, and BSA-NG 6) over a period of 2 h of investigation. In the first 15 min, the MBE was found to be in the range of 61.95–63.43%. After 120 min of observation, the MBE value increased to approximately 67%. In general, the MBE value trend increased according to the time of investigation, as illustrated in Figure 2b. The longer the interaction time, the higher the MBE value that was observed. Hence, this finding indicates an attractive interaction between BSA-NGs and mucin solution. In this experiment, the calculated free mucin from the preparation for each time point, measured after combining the formulation (BSA-NG 2, BSA-NG 4, and BSA-NG 6) with mucin, indirectly shows the magnitude of the interaction between the BSA molecule in the formulation and the added mucin solution. Such an interaction begins when the polymer chain becomes more flexible after being hydrated and starts to penetrate between the mucin molecules to initiate the mucoadhesion process [28,29,30]. Subsequently, the BSA-NGs firmly bind with the mucin, generating mucin-BSA complexes. It has been previously reported that BSA-NGs exhibit a higher degree of interaction with mucin at a neutral pH, possibly due to the attraction toward the mucin’s cysteine domain [26].

### 2.4. Thermal Gravimetry (TG) Analysis

**Figure 3:**
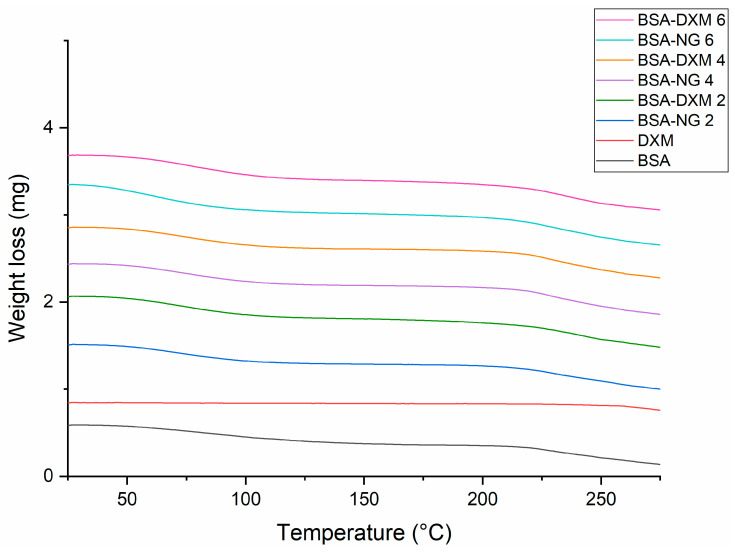
TG curves of BSA-NGs and the corresponding DXM-loaded nanogel formulations, as well as the initial components.

TG analysis was performed to determine the residual ethanol content and support the Raman investigations. The TG thermograms revealed that BSA undergoes denaturation above 60 °C, as indicated by the weight loss (Figure 3). Ethanol evaporates within the same temperature range (45–110 °C); therefore, we investigated the weight loss in the mentioned temperature interval and compared it with the TG curve of the initial BSA. As ethanol belongs to the Class 3 solvents, its residual concentration should be less than 5000 ppm in a daily dose of the final product, a requirement of the International Conference for Harmonization (ICH) Q3C (R5) guidelines for residual solvents [31]. It has been revealed that, when comparing the weight loss of BSA-NGs and corresponding DXM-loaded nanogel formulations to the initial BSA, no remarkable difference was obtained, indicating that the residual ethanol content is under the limit of detection, thereby meeting the acceptance criteria of the ICH (Table 2).

### 2.5. Differential Scanning Calorimetric (DSC) Analysis

**Figure 4:**
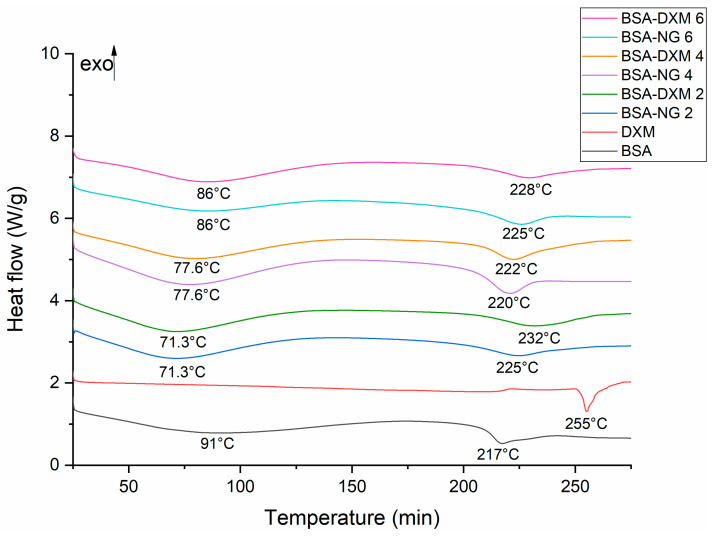
DSC thermograms of the BSA-NGs and the corresponding DXM-loaded nanogel formulations, as well as of the initial components.

DSC study was conducted in order to further investigate the thermal behavior of nanogel formulations (Figure 4). In accordance with the TG results, the endothermic peak at 91 °C that was seen in the DSC thermogram of the initial BSA indicates the denaturation temperature of protein, while the endothermic peak at approximately 217 °C belongs to its melting point [32]. The DSC curve of the DXM revealed that the drug has a melting point of 255 °C. In the case of nanogel formulations, the denaturation peak of BSA was markedly shifted to a lower temperature proportional to the BSA content of formulations [33]. Furthermore, no endothermic melting peak of DXM was observed in the case of DXM-loaded nanogel formulations, which indicated that the DXM was dispersed in a molecular state within the nanoparticles, either in an amorphous or in a disordered crystalline state [34]. However, the melting point of BSA increased in the corresponding DXM-loaded nanogel formulations, supporting the existence of the drug in the composition and the formation of a drug–albumin complex.

### 2.6. Morphological Characterization

**Figure 5:**
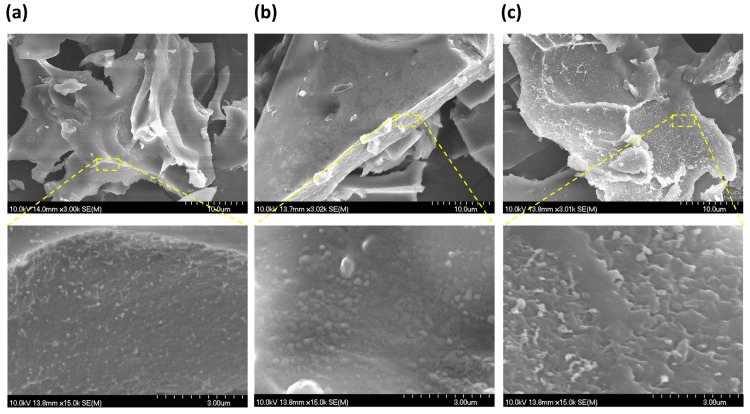
SEM images of the DXM-loaded nanogel formulations at different magnifications recorded form the surface marked with yellow square: BSA-DXM 2 (**a**), BSA-DXM 4 (**b**), and BSA-DXM 6 (**c**).

The morphology of the freeze-dried DXM-loaded nanogel formulations was investigated via SEM (Figure 5). The freeze-drying resulted in flake-like aggregates with a relatively flat, homogeneous, and smooth surface, while the cross-section of the flakes showed a dense structure with small-sized pores (as indicated by the yellow circles). Moreover, hydrogen bonding and hydrophobic interactions between the BSA and DXM may also have led to this type of porous skeleton structure.

### 2.7. Viscosity Measurement

**Figure 6:**
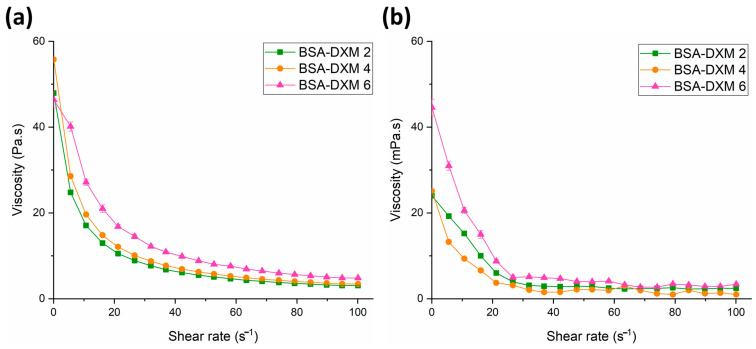
Viscosity curves of the DXM-loaded freshly prepared BSA-NGs preparations (**a**) and the corresponding redispersed form after the freeze-drying process (**b**).

For the viscosity (η) measurement, a non-linear relationship was obtained via steady-state rheological measurement as a function of the shear rate γ (s^−1^). As shown in Figure 6, the viscosity of all DXM-loaded BSA-NGs (BSA-DXM 2, BSA-DXM 4, and BSA-DXM 6) decreased as the shear rate increased from 0 to 100 s^−1^, indicating a non-Newtonian pseudoplastic flow with shear thinning behavior. This presented characteristic is desirable in nasal formulations since the pseudoplastic flow can potentially maintain drug deposition in the nasal cavity upon delivery through a nasal spray; hence, it might improve the retention time and prevent the formulation from rapid elimination due to nasal mucociliary clearance, which, in turn, can enhance drug absorption through the nasal mucosa [35,36].

Moreover, the viscosity mean value at 100 s^−1^ of the DXM-loaded BSA-NGs preparations, both before and after the freeze-drying process, exhibited a significant reduction in value that was around a thousand-fold decrease, as shown in Table 3. This phenomenon might occur due to the evaporation of the ethanol content during the lyophilization process. In addition, according to the results, the viscosity of the DXM-loaded BSA-NGs was found to be suitable for nasal liquid preparation as nearly all the preparations exhibit a moderate viscosity, a property that can potentially improve the residence time in the nasal mucosa [37,38].

### 2.8. Drug Release Studies

**Figure 7:**
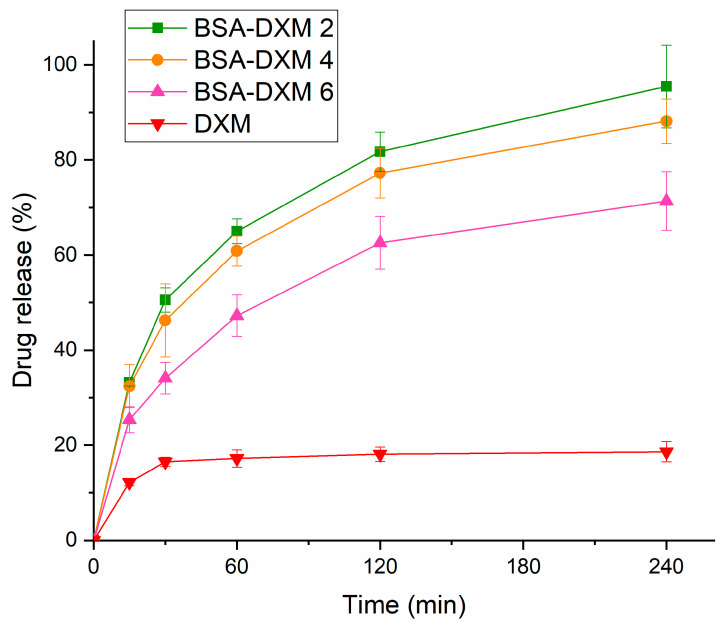
The in vitro drug release profile of the DXM-loaded BSA-NGs.

Drug release studies were conducted by employing an SNES medium to mimic the conditions in the nasal cavity. The results revealed that all the DXM-loaded BSA-NGs preparations (BSA-DXM 2, BSA-DXM 4, and BSA-DXM 6) showed increased drug release within four hours of investigation (Figure 7), compared to the pure DXM.

In the first hour, the release amount was found to be more than 60% for the preparations of BSA-DXM 2 and BSA-DXM 4. Meanwhile, for the preparation of BSA-DXM 6, the release amount was about 47.24%. The pure DXM showed a lower value, which represented no more than 20% of drug release. Furthermore, after four hours, the amount of drug release for BSA-DXM 2 and BSA-DXM 4 were found to be higher, about 95.47% and 88.15%, respectively. At the same time, the release amount of BSA-DXM 6 was approximately 71.36%. The release amount of BSA-DXM 6 appeared lower compared to the BSA-DXM 2 and BSA-DXM 4 preparations, which could possibly be due to the higher viscosity of BSA-DXM 6 than the other formulations, hindering the drug’s release from the gel base [18]. In line with this hypothesis, the release amount of pure DXM that was observed when it was in steady-state conditions remained unchanged. This could be due to the poor solubility of DXM in water, thereby reducing the release of the drug into the medium.

This finding indicates that the BSA-NGs prepared through the ethanol-induced gelation method can achieve a prolonged drug release profile in simulated nasal conditions, which is also promoted by appropriate viscosity behavior. Interestingly, the release profiles of the BSA-NGs provide an illustration regarding the possible release profile of a drug when incorporated into BSA-NGs for nasal route application. Accordingly, this illustration might be useful in a future drug development process in which this application can be considered for nasal drug delivery, such as nasal delivery to achieve brain targeting, systemic therapy, or local therapy [7].

## 3. Conclusions

The preparation of BSA-NGs by utilizing the ethanol-induced gelation method showed promising characteristics for application in the nasal passages. According to the investigations and by taking into account the conditions of the nasal cavity, it was found that the composition of albumin, ethanol, and water plays an essential role in the properties of the prepared BSA-NGs. Moreover, the pH measurement revealed a suitable pH value for the nasal environment, promoting the non-irritating properties of the BSA-NGs. The investigation of mucoadhesion behavior through ZP changes and the turbidimetric method highlighted an attractive interaction between the BSA-NGs and mucin solution, seemingly opening up the possibility of avoiding rapid clearance due to the nasal mucociliary mechanism.

Furthermore, the Raman spectrum confirmed that there was no change in the secondary structure of BSA, and no ethanol remained in the sample after the freeze-drying process, as confirmed by the DSC and TG results. Additionally, investigation of the drug-loaded BSA-NGs showed that the preparation exhibited moderate viscosity, facilitating remarkable drug release from the BSA gel base in nasal conditions. Collectively, these results may illustrate the potential of BSA as a delivery carrier that can be utilized for further drug development, especially for nasal delivery purposes.

## 4. Materials and Methods

### 4.1. Chemicals

BSA (lyophilized powder, purity ≥ 97%), ethanol 96% *v*/*v* DXM was used as a model drug (Merck, Ltd., Budapest, Hungary), along with mucin from the porcine stomach (type III) and simulated nasal electrolyte solution (SNES), which consisted of 2.98 g/L of potassium chloride (KCl), 8.77 g/L of sodium chloride (NaCl), and 0.59 g/L of anhydrous calcium chloride (CaCl_2_), dissolved in purified water (pH 5.6). All other reagents were purchased from Merck Ltd. (Budapest, Hungary) unless indicated otherwise. Analytical-grade methanol was purchased from Molar Chemicals (Budapest, Hungary). Purified water was obtained using a gradient water purification system (Millipore Milli-Q^®^, Merck Ltd., Budapest, Hungary). All reagents and solvents were of pharmaceutical grade.

### 4.2. Optimization of BSA-NGs

The preparation of ethanol-induced BSANGs was optimized using a 3-factor, 3-level full factorial design in 9 independent experiments. TIBCO Statistica^®^ 13.4 (Statsoft Hungary, Budapest, Hungary) software was utilized to generate the design of the experiment. The amounts of BSA 20% *w*/*v* (*x*_1_), ethanol (*x*_2_), and purified water (PW) (*x*_3_) were selected as the independent variables, and the effect was observed at low, medium, and high levels, as depicted in Table 4.

In this experiment, the concentration of BSA solution used was 20% *w*/*v*, in order to ensure enough BSA concentration for the formation of intermolecular secondary structure (β-sheet) [18]. Also, the utilized amount of BSA solution and ethanol was considered a critical parameter in the BSA nanogelation process, which requires 3 mM of BSA with a minimum of 0.4 mL of ethanol [18,39]. Therefore, the amount of those variables was chosen to be within the above-mentioned concentrations, along with the necessity for PW to perform the gelation process. In brief, the BSA 20% *w*/*v* solution was set to be from 0.5 to 1.5 mL, while the content of ethanol ranged from 0.6 to 1.2 mL, and the amount of PW was from 0.1 to 0.9 mL. Moreover, the particular design of the experiment was employed to investigate the quadratic response surface and to calculate the relationship between variables, using the following second-order polynomial model:

*Y* = *β*_0_ + *β*_1_*x*_1_ + *β*_11_*x*_1_^2^ + *β*_2_*x*_2_ + *β*_22_*x*_2_^2^ + *β*_3_*x*_3_ + *β*_33_*x*_3_^2^ (3)

where *Y* is the response variable; *β*_0_ is a constant; *β*_1_, *β*_2_, and *β*_3_ are linear coefficients; *β*_11_, *β*_22_, and *β*_33_ are quadratic coefficients; *x*_1–3_ are the main effect factors; and *x*_1_^2^, *x*_2_^2^, and *x*_3_^2^ are the quadratic effect factors. The effect of the factors on the dependent variables (Z-average, PdI, and ZP) at 25 °C was presented statistically as the response surface model and analysis of variance (ANOVA) with a 95% confidence interval level, and the variable was considered significant if *p* < 0.05.

### 4.3. Preparation of BSA-NGs

BSA-NGs were prepared via the desolvating method [10,15,16], using a composition ratio according to the experimental design results. Briefly, a specified amount of BSA 20% *w*/*v* (0.5–1.5 mL) was added to purified water (0.1–0.9 mL) under constant stirring (500 rpm, 37 °C). Then, a certain amount of ethanol (0.6–1.2 mL) was added dropwise. After that, the mixture was homogenized with constant stirring (500 rpm, 37 °C) using a hot-plate magnetic stirrer until the gelation process finished, in which the formation of a high-viscosity solution, a bluish-soft gel, and a hard gel could be observed for each formulation. Afterward, all the prepared formulations were transferred into vials and then lyophilized using a freeze-dryer (ScanVac CoolSafe, LaboGene, Lynge, Denmark) for 16 h (−40 °C, 0.013 mbar of pressure), and then continued with secondary drying for 4 hours at 25 °C. Subsequently, all the freeze-dried cakes were stored in the fridge at 4 °C for further analysis and were immediately reconstituted with a specific amount of purified water prior to each investigation.

Furthermore, in order to study the viscosity behavior and the drug release profile, a model drug (DXM) was incorporated into the BSA-NGs formulation. An accurately weighed amount of 5 mg DXM was employed to prepare DXM-loaded BSA-NGs using the same procedure, with DXM that was previously suspended in various amounts of water and BSA, according to the required composition.

### 4.4. Characterization of BSA-NGs

#### 4.4.1. Dynamic Light Scattering Measurements

The measurement of the Z-average, PdI, and ZP (ζ) of the reconstituted BSA-NGs was carried out via the dynamic light scattering (DLS) method, using a Malvern Zetasizer Nano ZS (Malvern Instruments, Worcestershire, UK) instrument. All the freeze-dried cakes of BSA-NGs were redispersed with 1.5 mL of PW and stored in folded capillary cells at 25 °C. All measurements were carried out in triplicate (*n* = 3) and the results were expressed as the average ± SD.

#### 4.4.2. pH Measurement

The pH of all the BSA-NGs preparations was measured after reconstitution with a certain amount of purified water, using a pH tester (WTW^®^ inoLab^®^ pH 7110 laboratory, Thermo Fisher Scientific, Budapest, Hungary). All measurements were conducted in triplicate (*n* = 3). The results were presented as means ± SD.

#### 4.4.3. Raman Spectroscopy

For Raman spectroscopic analysis, the BSA-NG 2, BSA-NG 4, and BSA-NG 6 preparations were employed, based on the differences in their appearance after the gelation process. A Thermo Fisher DXR dispersive Raman microscope (Thermo Fisher Scientific Inc., Waltham, MA, USA) was utilized to assess the changes in the secondary protein structure of BSA-NGs at the preparation step. The instrument was equipped with a CCD camera and a diode laser running at a 780 nm wavelength, with a laser power of 12 mW and a 50-µm slit aperture size. The obtained spectrum was recorded within a 2-second exposure time and a 6-second acquisition time, collecting a total of 32 scans per spectrum in the spectral range of 3300–200 cm^−1^, including the cosmic ray and fluorescence corrections.

#### 4.4.4. Mucoadhesive Properties of BSA-NGs

The mucoadhesive properties of all BSA-NGs preparations were investigated by studying the interaction with mucin using two different methods: the turbidimetric (direct) method and ZP measurement (indirect method). For the turbidimetric method, an equal volume of reconstituted BSA-NG preparation and mucin solution (0.5% *w*/*v* in SNES) were mixed and incubated at 37 °C for 2 h. Afterward, at predetermined time intervals of 15, 30, 60, and 120 min, the mixtures were then centrifuged at 16,000 rpm for 10 min (4 °C). After that, the supernatant content (free mucin) was quantified using a Jasco V-730 UV spectrophotometer (ABL&E-JASCO Ltd., Budapest, Hungary) at 255 nm. The mucin binding efficiency (MBE) of the BSA-NG preparations was then calculated following the equation below [40,41,42,43,44,45,46,47]:

(4) $$Mucin\;binding\;efficiency\;\left(\%\right)=\frac{Total\;mucin\;used-Free\;mucin}{Total\;mucin\;used}\;\times \;100$$

Furthermore, the mucoadhesive properties of the BSA-NG preparations were also investigated, using the Malvern Nano ZS instrument (Malvern Instruments, Worcestershire, UK) by measuring the changes in zeta potential value that take place due to the interaction of negatively charged mucins with the BSA-NG preparations [48,49,50].

#### 4.4.5. Thermal Gravimetry

The TG investigation was performed with a METTLER-Toledo TGA/DSC 1 (Mettler-Toledo GmbH, Gießen, Germany) instrument. Samples of approximately 10 mg were measured into 100 µL aluminum pans, which were then crimped and inserted into the furnace. The experiment was conducted in the temperature range of 25 °C to 275 °C, with a 10 °C/min heating rate, under a constant nitrogen flow of 50 mL/min. The results were evaluated using the STARe software version 9.00.

#### 4.4.6. Differential Scanning Calorimetry

The DSC examinations were conducted using a Mettler-Toledo DSC 1 (Mettler-Toledo GmbH, Gießen, Germany) instrument at a temperature interval of 25 to 275 °C, with a heating rate of 10 °C/min, under a constant argon flow of 150 mL/min. For the experiments, approximately 5 mg of the samples were measured into 40 µL aluminum pans. Each measurement was normalized to the sample size. The results were evaluated using the STARe software version 9.00.

#### 4.4.7. Scanning Electron Microscope

The morphological characterization of the DXM-loaded nanogels was investigated using an SEM (Hitachi S4700, Hitachi Scientific Ltd., Tokyo, Japan) at 10 kV. The samples were coated with gold–palladium (90 s) using a sputter coating apparatus (Bio-Rad SC 502, VG Microtech, Uckfield, UK), in order to induce electric conductivity on the surface of the samples.

#### 4.4.8. Viscosity Studies

The viscosity studies were carried out with selected BSA-NGs loaded with DXM as the model drug. The measurement was conducted using a RheoStress 1 HAAKE rheometer instrument (Thermo Fisher Scientific, Karlsruhe, Germany). A thermostatic circulator (Thermo Haake Phoenix II + Haake C25P) was connected to the equipment to ensure a constant temperature during the measurement. The measurements were conducted using cone–plate geometry (gap: 0.052 mm, radius: 49.9 mm, angle: 1°) and the apparent viscosity of the samples was observed over a shear rate sweep of 0.01–100 s [26,27,28]. The measurements were performed with the Haake RheoWin^®^ Job Manager v.3.3 software (Thermo Electron Corporation, Karlsruhe, Germany). After that, the collected data were analyzed with the Haake RheoWin^®^ Data Manager v.3.3 software (Thermo Electron Corporation, Karlsruhe, Germany). All tests were carried out in triplicate at 35 ± 0.5 °C (*n* = 3) and the results were expressed as the average ± SD.

#### 4.4.9. In Vitro Drug Release Studies

To investigate the in vitro drug release behavior of different BSA-NGs loaded with DXM as a model drug, the modified paddle method was employed in a Hanson SR8-Plus^TM^ dissolution tester (Teledyne Hanson Research, Chatsworth, CA, USA). Briefly, 1 mL of DXM-embedded BSA-NG preparations were stored in a dialysis bag with MWCO of 12–14 kD (Spectra/Por^®^, Spectrum Labs, San Francisco, CA, USA) and then immersed in 100 mL of freshly prepared SNES to act as the release medium. The investigation was carried out at 50 rpm (35 ± 0.5 °C) and an aliquot of 0.5 mL of the released medium was withdrawn at 15, 30, 60, 120, and 240 min. After that, the amount of DXM released was analyzed using the HPLC method, as described below. Three parallel measurements (*n* = 3) were performed, and the results were presented as means ± SD.

#### 4.4.10. HPLC Analysis

The concentration of DXM released from the BSA-DXM preparations was quantified using an Agilent Infinity 1260 HPLC (Agilent Technologies, Santa Clara, CA, USA). A Gemini^®^ NX 5u C18 110A column (150 mm × 4.6 mm, 5 µm (Phenomenex, Torrance, CA, USA)) was used as the stationary phase. Isocratic elution was performed for 10 min with a mobile phase consisting of PW (A) and acetonitrile—methanol 50:50 solution (B) applied in a 55:45 ratio. The analysis was carried out at 40 °C, with a 10 µL injection volume and a 1 mL/min flow rate. The concentration of DXM was determined using a UV-VIS diode array detector (254 nm) and was processed using Chem-Station B.04.03 software (Agilent Technologies, Santa Clara, CA, USA). The regression coefficient (R^2^) was 0.999 within a concentration range of 0.5–50 µg/mL for the calibration curve, with a retention time of 7.57 min. The limit of detection (LOD) and the quantification (LOQ) of DXM were acquired at 15 ppm and 44 ppm, respectively.

## Figures and Tables

**Table 1:** Composition of the experimental design with the observed responses. Data are presented as means ± SD.

Batch	Independent Variables			Z-Average ± SD (nm)	PdI ± SD	ZP ± SD (mV)	Appearance after Gelation
	BSA 20% *w*/*v* (mL)	Et-OH (mL)	PW (mL)				
BSA-NG 1	0.5	0.6	0.1	100.63 ± 9.12	0.687 ± 0.16	−32.2 ± 0.64	Hard gel, turbid
BSA-NG 2	0.5	0.9	0.9	77.55 ± 0.51	0.409 ± 0.02	−29.7 ± 0.40	High viscosity, bluish
BSA-NG 3	0.5	1.2	0.5	81.81 ± 3.68	0.297 ± 0.01	−33 ± 0.60	Hard gel, turbid
BSA-NG 4	1.0	0.6	0.9	64.67 ± 2.64	0.326 ± 0.01	−9 ± 0.02	Liquid, clear
BSA-NG 5	1.0	0.9	0.5	71.51 ± 13.45	0.584 ± 0.01	−19.2 ± 0.37	Hard gel, turbid
BSA-NG 6	1.0	1.2	0.1	138.6 ± 7.43	0.418 ± 0.01	−29.1 ± 0.35	Hard gel, turbid
BSA-NG 7	1.5	0.6	0.5	64.35 ± 6.12	0.404 ± 0.04	−10.8 ± 3.44	Liquid, clear
BSA-NG 8	1.5	0.9	0.1	198.7 ± 3.1	0.479 ± 0.01	−31.8 ± 0.45	Hard gel, turbid
BSA-NG 9	1.5	1.2	0.9	98.91 ± 5.78	0.675 ± 0.01	−15.6 ± 1.33	Liquid, clear

**Table 2:** Weight-loss results for the BSA-NGs and the corresponding DXM-loaded nanogel formulations in comparison to the initial BSA.

Formulation	Weight Loss (mg)	Weight Loss (%)
initial BSA	0.2507	6.67
BSA-NG 2	0.2515	6.69
BSA-DXM 2	0.2499	6.65
BSA-NG 4	0.2526	6.72
BSA-DXM 4	0.2511	6.68
BSA-NG 6	0.2567	6.83
BSA-DXM 6	0.2548	6.79

**Table 3:** Viscosity mean value at 100 s^−1^ of the DXM-loaded BSA-NGs before and after the freeze-drying process.

Formulation	Before Freeze-Drying η (Pa.s) ± SD	After Freeze-Drying η (mPa.s) ± SD
BSA-DXM 2	3.091 ± 0.038	2.451 ± 0.034
BSA-DXM 4	3.442 ± 0.015	0.997 ± 0.031
BSA-DXM 6	4.846 ± 0.199	3.396 ± 0.156

**Table 4:** Design of experiment for BSA-NGs optimization, showing the selected independent variables with the 3-level (−1, 0, and +1) investigated values.

Factors	Level		
	−1	0	+1
BSA 20% *w*/*v* (mL)	0.5	1.0	1.5
Ethanol (mL)	0.6	0.9	1.2
PW (mL)	0.1	0.5	0.9

## Data Availability

The data presented in this study are openly available in article.

## Supplementary Materials

The following supporting information can be downloaded at: https://www.mdpi.com/article/10.3390/gels9110896/s1, Figure S1: Response surface plots showing the effect of independent variables PW—Ethanol (a), BSA—Ethanol (b) and PW—BSA (c) on Z-average as well as PW—Ethanol (d), BSA—Ethanol (e) and PW—BSA (f) on PdI.
