# Supplementary material for: Preparation and Optimization of Bovine Serum Albumin Nanoparticles as a Promising Gelling System for Enhanced Nasal Drug Administration
Source: Gels. 2023 Nov 13;9(11):896. doi: 10.3390/gels9110896 (PMC10670644; doi:10.3390/gels9110896)
Supplement: Supplementary file 1 [file gels-09-00896-s001.zip › gels-2679538-supplementary.pdf]

Supplementary material

Preparation and optimization of bovine serum albumin nano-particles as a promising gelling system for enhanced nasal drug administration

Sandra Aulia Mardikasari 1,2, Gábor Katona 1,*, Bence Sipos 1, Rita Ambrus1 and Ildikó Csóka 1

[1] Institute of Pharmaceutical Technology and Regulatory Affairs, Faculty of Pharmacy, University of Szeged, Eötvös St. 6, H-6720 Szeged, Hungary; sandraaulia@unhas.ac.id (S.A.M.); katona.gabor@szte.hu (G.K.); sipos.bence@szte.hu (B.S.); ambrus.rita@szte.hu (R.A.); csoka.ildiko@szte.hu (I.C.)

[2] Faculty of Pharmacy, Hasanuddin University, Makassar 90245, Indonesia; sandraaulia@unhas.ac.id (S.A.M.)

* Correspondence: katona.gabor@szte.hu (G.K.)

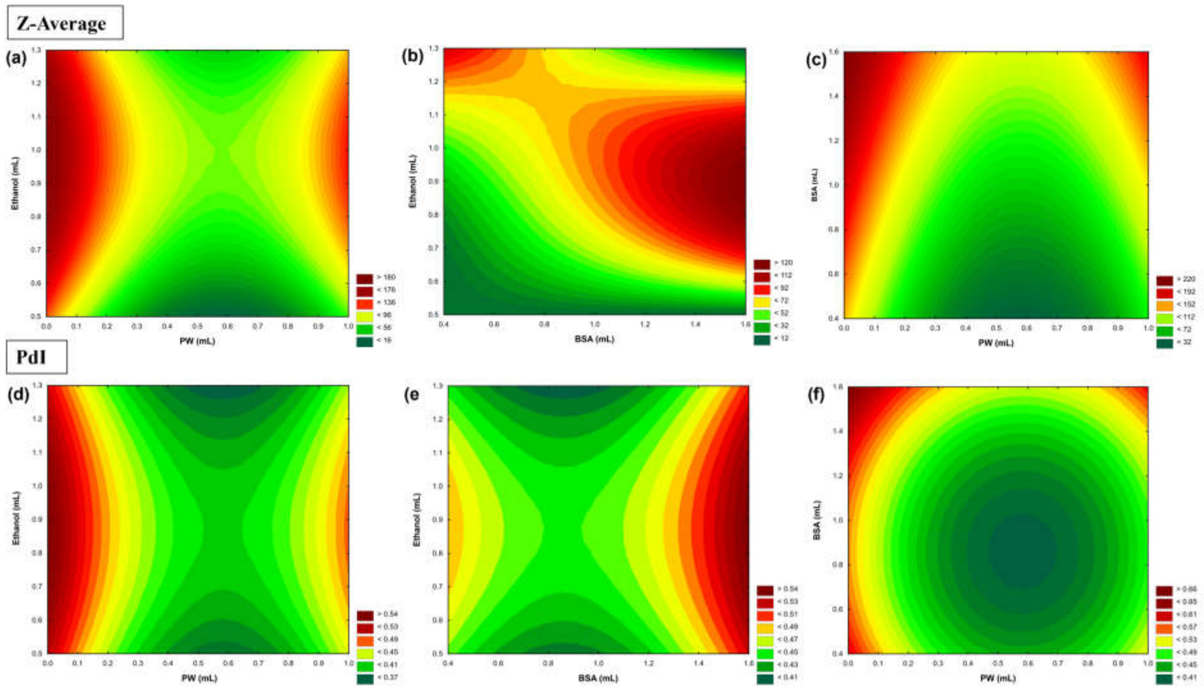

Figure S1. Response surface plots showing the effect of independent variables PW — Ethanol (a), BSA — Ethanol (b) and PW — BSA (c) on Z-average as well as PW — Ethanol (d), BSA — Ethanol (e) and PW — BSA (f) on PdI.
